# Réplica à carta ao editor referente ao artigo: “Efeitos da infiltração intra-articular de aspirado de medula óssea no tratamento da osteoartrite de joelho: Estudo clínico comparando BMA *versus* corticosteroide e bloqueio genicular”. *Rev Bras Ortop* 2025;60(2):s00451809512. DOI: 10.1055/s-0045-1809512

**DOI:** 10.1055/s-0046-1817132

**Published:** 2026-03-19

**Authors:** Renata Clazzer, Dilamar Moreira Pinto, Mariana Valois de Aquino Krause, Tale Lucas Vieira Rolim, Ricardo Lyra de Oliveira, Diego Ariel de Lima

**Affiliations:** 1 Departamento de Ortopedia e Traumatologia, Hospital Otavio de Freitas, Recife, PE, Brasil; 2 Departamento de Ciências da Saúde, Centro de Ciências Biológicas e da Saúde, Universidade Federal Rural do Semi-Árido, Mossoró, RN, Brasil

Prezado Editor, Dr. Geraldo Rocha Motta Filho,

Agradecemos aos autores da carta referente ao nosso artigo publicado na *Revista Brasileira de Ortopedia* 1 pelas observações apresentadas, que consideramos pertinentes e construtivas. O debate científico, quando conduzido com respeito e embasamento técnico, é essencial para o amadurecimento da ortobiologia como campo de pesquisa e prática clínica. Concordamos plenamente que os resultados devem ser interpretados com cautela, e que a busca por rigor científico e clareza conceitual é fundamental. Valorizamos, portanto, o espírito colaborativo deste debate, que certamente contribuirá para o avanço responsável das terapias ortobiológicas no manejo da osteoartrite de joelho.

## Respostas aos Três Pontos Questionados

1. Aspectos Metodológicos
  Reconhecemos as limitações apontadas, incluindo a perda de seguimento de 15 pacientes, o que reduziu o poder estatístico. Essa limitação foi claramente descrita no artigo.
O número inicial (25 por grupo) baseou-se em estudos semelhantes. 2 Considerando diferença esperada de 20% no Western Ontario and McMaster Universities Arthritis Index (WOMAC) e Alfa de 5%, o cálculo amostral indicaria aproximadamente 25 a 35 pacientes por grupo para manter poder de 80%. **Reconhecemos que, para alcançar o poder inicialmente planejado, seria desejável uma amostra maior, já estimando as possíveis perdas de seguimento e a reposição dos casos perdidos** . No entanto, optamos por não ampliar a inclusão devido a restrições logísticas e financeiras, e descrevemos essa limitação de forma honesta e explícita.
2. Confusão conceitual sobre a intervenção
Agradecemos a observação sobre o uso de dexametasona. Esclarecemos que sua inclusão visou apenas padronizar o protocolo clínico previamente adotado em nossa instituição, também aplicado ao grupo de controle. A dose intra-articular empregada (≈ 0,8 mg) foi inferior à usual faixa na qual há relatos de toxicidade celular (2–4 mg). 3 Ademais, a dexametasona é um corticosteroide de curta duração, com meia-vida plasmática de 3 a 5 horas e duração biológica de até 72 horas, o que minimiza o risco de exposição prolongada das células-tronco mesenquimais (CTMs), diferindo dos corticosteroides de depósito, como triancinolona e betametasona, de liberação lenta e maior potencial citotóxico.
Há relatos *in vitro* de que glicocorticoides em altas concentrações e/ou exposições prolongadas reduzem a viabilidade e a diferenciação condrogênica das CTMs. Todavia, tais efeitos são dependentes de dose e tempo, e não representam consenso de toxicidade universal. Em contrapartida, há evidência experimental de que a dexametasona em baixas doses estimula a condrogênese e potencializa o fator de transformação do crescimento Beta ( *transforming growth factor Beta* , TGF-β, em inglês), conforme descrito no próprio artigo citado pelos autores da carta ao editor (Csaki et al., 4 2008):
  “Dexamethasone, a synthetic glucocorticoid, stimulates chondrogenesis directly via the glucocorticoid receptor […]. Interestingly, dexamethasone potentiates the chondrogenic stimulation of TGF-β.”
O artigo de Csaki et al. 4 não relata efeito deletério, tampouco dose tóxica, e descreve justamente o papel modulador e pró-condrogênico da dexametasona, a depender da dose.
Reconhecemos as controvérsias sobre o uso de dexametasona associada ao aspirado de medula óssea ( *bone marrow aspirate* , BMA, em inglês), mas optamos por manter o corticoide no protocolo justamente para preservar a similaridade entre os grupos e evitar que a ausência da dexametasona criasse uma variável adicional não controlada, mantendo como diferença isolada apenas o uso do BMA.
3. Interpretação dos resultados
  Agradecemos a observação referente à interpretação dos resultados. De fato, como corretamente apontado, nossos achados somente demonstraram diferença significativa no domínio “dor” do WOMAC aos 6 meses, sem diferença em rigidez ou função. Essa limitação foi reconhecida na conclusão e na discussão do artigo.
Quanto à publicação na página do Instagram da Sociedade Brasileira de Ortopedia e Traumatologia (SBOT; @sbotnacional; https://www.instagram.com/p/DOonWhxE20A/?igsh=MTltcW1xaWszcXA1eA== ), reconhecemos que a publicação simplificou os achados, e que a frase “melhora da dor e função” pode ser interpretada de modo mais amplo do que o texto científico original. Concordamos que o enunciado poderia ser mais preciso, embora entendamos tratar-se de uma comunicação resumida, que estimula a leitura completa do artigo na *Revista Brasileira de Ortopedia* .
